# A Turing test for collective motion

**DOI:** 10.1098/rsbl.2015.0674

**Published:** 2015-12

**Authors:** J. E. Herbert-Read, M. Romenskyy, D. J. T. Sumpter

**Affiliations:** Department of Mathematics, Uppsala University, Uppsala 75106, Sweden

**Keywords:** collective motion, Alan Turing, citizen science

## Abstract

A widespread problem in biological research is assessing whether a model adequately describes some real-world data. But even if a model captures the large-scale statistical properties of the data, should we be satisfied with it? We developed a method, inspired by Alan Turing, to assess the effectiveness of model fitting. We first built a self-propelled particle model whose properties (order and cohesion) statistically matched those of real fish schools. We then asked members of the public to play an online game (a modified Turing test) in which they attempted to distinguish between the movements of real fish schools or those generated by the model. Even though the statistical properties of the real data and the model were consistent with each other, the public could still distinguish between the two, highlighting the need for model refinement. Our results demonstrate that we can use ‘citizen science’ to cross-validate and improve model fitting not only in the field of collective behaviour, but also across a broad range of biological systems.

## 1. Introduction

Alan Turing provided a means of assessing whether a machine's behaviour was equivalent or indistinguishable from that of a human [1]. In the Turing test, if a human observer could not determine between which one of two interacting players was a machine (the other a human), then the machine had passed the test and exhibited intelligent behaviour. The test is designed to assess the ability of a model (the machine) to reproduce the real world (human behaviour).

While the design of a machine that accurately simulates a human is still some way off, models of other aspects of animal behaviour are becoming increasingly realistic [2,3]. The collective motion of animal groups provides one key example. Bird flocks and fish schools move together using local interaction rules whereby they respond to the movements and positions of their neighbours [4]. Literally thousands of models, each with slight variations on a theme, have been proposed to explain these phenomena [4]. In terms of quantity, data collected on the movements of real animal groups lag behind the theoretical models [5–7]. Nevertheless, these data have been used to generate models aimed at explaining how individuals in groups interact using simple rules, and how these rules reproduce the collective properties of swarms, flocks and schools [6,8–10]. The large-scale statistical properties of these simulations, such as a group's order and structure, often match those of real fish schools or bird flocks [6,8].

The recognized method for validating models is through statistical comparison of data and model [11]. These comparisons can be made both at the level of the individual and the collective [12]. However, on a daily basis, biologists and modellers adopt an approach much more similar to that proposed by Turing. We run our simulation model, look at its output and compare it to real animal movement. This practice raises two important questions. If the statistical properties of some data adequately match those simulated by a model, but the model does not ‘look’ correct, should we be satisfied with our model? If not, how can we formalize an observational test so that we can be satisfied our model reproduces the data?

To address this question, we first collected data on the movements of real fish schools. We then followed a standard procedure for fitting a collective behaviour model to these data [6,13]. We then developed an online game where people were asked if they could distinguish between the movements of real fish or those simulated by a model. We asked whether the model, even if it statistically captured the properties of fish schools, could be distinguished from the movements of real fish schools. In essence, this is a Turing test designed to assess whether a model can accurately mimic the properties of a biological system. Indeed, Harel [14] and Cronin *et al*. [15] previously proposed that Turing-like tests could be used to assess the ability of biological models to simulate real life [14,15]. They suggested that if a model of some animal or cell could not be distinguished from a real animal or cell, then the model had passed the test and captured some properties of the biological system in question [14,15]. Here, we implement these ideas by assessing whether a model of collective motion can capture the movements of schooling fish.

## 2. Material and methods

Pacific blue-eyes (*Pseudomugil signifer*) were caught in hand nets from Narrabeen Lagoon, New South Wales, Australia (33°43′03 S, 151°16′17 E). Fish were kept in filtered freshwater in 150 l glass tanks at 22–25° and fed crushed flake food *ad libitum*. Fish were housed for at least three weeks prior to experimentation. The experimental arena was circular (diameter = 760 mm) and filled to a depth of 70 mm with aged and conditioned tap water. The arena was lit by fluorescent lamps and was visually isolated. For each trial, we randomly selected *N* fish (*N* = 10, 20, 30, 40, 50 or 60) of similar size (approx. either 7.5 or 13 mm) from the housing tanks and placed them in the experimental arena (see the electronic supplementary material, table S1 for details of trial numbers). Fish were left to acclimate to the arena for at least 5 min, after which they were filmed for 15–20 min at 15 frames per second using a camera (Logitech Pro 9000) placed directly above the centre of the arena. Using automated tracking software [16], we recorded the movements and positions of the fish.

We used these data to inform a model of collective motion. Our model was a self-propelled particle model adapted from Vicsek *et al*. [17] and refined using data collected from the real fish. We compared two major statistical properties of the real fish schools with simulations of our model: polarization and nearest-neighbour distance (NND; see the electronic supplementary material for a full description of the model and details of these calculations). Polarization turns zero when the fish/particles are completely disordered and assumes finite positive values, with a maximum of 1, when the fish/particles are completely aligned. The NND was computed by comparing the spatial position of the focal fish (or particle) with positions of other individuals. All statistics were calculated for every fish/particle on every frame and every video. For the simulations, the statistics were collected when the simulation reached a steady state, averaged over five independent runs.

We then designed an observational test to see if people could distinguish between the movements of real fish or simulated data. The test consisted of showing two videos in adjacent windows. In one window, we showed dots moving in the same trajectories as the tracks from a real fish school (recorded in our experiments). In the other window, we showed dots moving in trajectories that were generated from our simulation model. We used just one group size (*n* = 10 fish/particles) to avoid changing group size between players. Each player was shown six pairs of windows and was asked to select the video of the real fish (and not the simulated one) in each pair. We first gave the test to a group of biologists and theoreticians (18 people) who research collective motion. We then made the game available online (http://www.collective-behavior.com/apps/fishgame/) and advertised it through Twitter. We assessed whether experts and members of the public could distinguish between the movements of real fish and the movements of simulated fish schools.

## 3. Results

**Figure 1.**
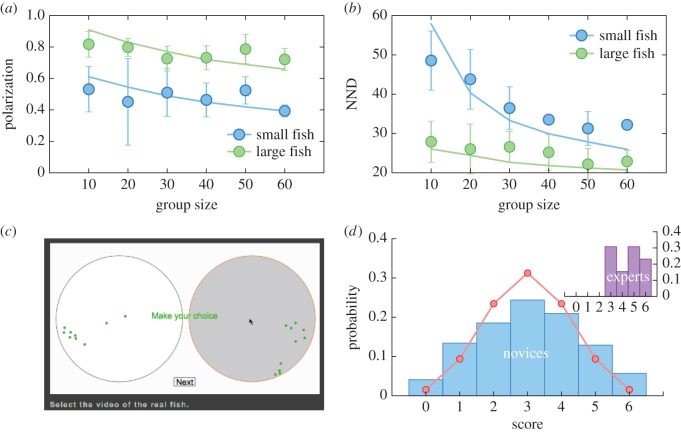
Comparison of statistical properties in experiment and simulations, game interface and results of the test. (*a*) Average polarization ± 1s.d. and (*b*) NND ± 1s.d. as a function of group size. Lines correspond to simulations, while dots represent experimental results. (*c*) A screenshot of the web interface of the game. (*d*) Distributions of players' scores. The line in the main plot represents the expected binomial distribution. (Online version in colour.)

Small fish had lower polarization and higher NND than large fish, with NND decreasing with group size in both fish sizes (figure 1*a*,*b*). By changing one parameter in our model, namely the perception range over which individuals interacted, we could reproduce the difference between small and large fish. Our model also reproduced the change in polarization and NND distributions for both small and large fish, without any further changes in parameter values. The modelled perception range was smaller for smaller fish than for larger fish, providing a parsimonious biological interpretation of our results.

We then asked whether our simulations, even though they matched the statistical properties of the real fish schools, could be distinguished by human observers using the observational test (figure 1*c*). ‘Experts’ were successfully able to distinguish between the movements of the real fish and simulated ones on their first attempt at the game (figure 1*d* inset). We then made the game available online and asked members of the public to play. Results presented in the main plot in figure 1*d* are for 1775 players (based on the number of unique IP addresses). While members of the public could distinguish between the movements of real fish and those of simulated ones, they did not consistently choose the real fish; scores of 0 or 6 occurred more than expected by chance (*χ*^2^-test; *χ*^2^ = 367.7, d.f. = 6, *p* < 0.0001; figure 1*d*). In other words, they could tell the difference between the simulation and the real schools, but were unsure which was which.

**Figure 2.**
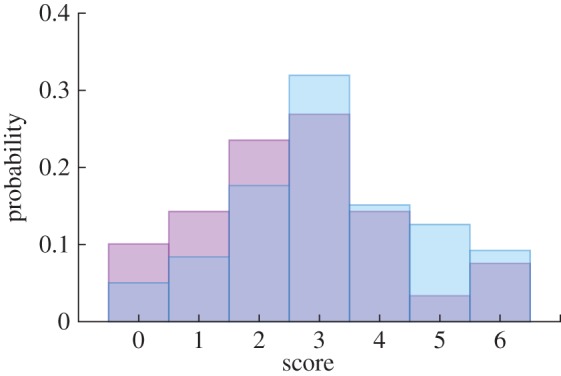
Distributions of online players' (*n* = 119) scores for the first (violet) and second (blue) attempts. (Online version in colour.)

We identified those online players that played the game more than once (*n* = 119). We then tested whether these players' scores increased on their second play of the game compared with their first play (figure 2). Individuals' scores significantly increased between their first and second play (paired *t*-test; *t* = −3.2, d.f. = 118, *p* = 0.002); they selected the real fish more often on the second attempt. In addition, players scores were correlated between their first and second play; individuals were consistent in whether they picked the real or simulated fish between plays (Pearson correlation, *R* = 0.24, *n* = 119, *p* = 0.009). Those online players that answered all six questions correctly were provided an opportunity to give feedback on how they differentiated between the real schools and the simulated ones. These players commonly suggested that the spatial organization of the groups and smoothness of the trajectories appeared different between the simulated and real schools.

## 4. Discussion

Our results highlight a limitation in fitting detailed models to real-world data. While large-scale statistical properties of a system might be captured by a model, detailed differences between simulations and real-world data can still be identified. In addition to the development of new techniques to cross-validate model fitting at a range of scales [12], observational tests like the one proposed here are relatively straightforward to implement, and could be used to cross-validate models. These would provide a valuable alternative to the standard methods of least-squares or maximum-likelihood fitting. Because players were better at selecting the real fish on their second attempt of the game, we can even envisage using this technique to evolve the parameters of a model, allowing game players to progress to new levels only when they correctly identify the difference. In addition, feedback from players could provide useful information to address weaknesses in different aspects of the models. Techniques that use public interest in science to improve models [18], and inspired by Turing's original insight, should provide a way of understanding the dynamics of other complex systems and other forms of biological imitation.

## Supplementary Material

Supplementary Text

## Data accessibility

The data underlying this study are available from Dryad: http://dx.doi.org/10.5061/dryad.d449r.
