# Supplementary material for: A Turing test for collective motion
Source: Biol Lett. 2015 Dec;11(12):20150674. doi: 10.1098/rsbl.2015.0674 (PMC4707694; doi:10.1098/rsbl.2015.0674)
Supplement: Supplementary Text [file rsbl20150674supp1.pdf]

A Turing Test for collective motion
Supplementary Material

Herbert-Read, J. E.,[1,*] Romenskyy, M.,[1,*] and Sumpter, D.J.T.[1,*]
[1]*Department of Mathematics, Uppsala University, 75106, Uppsala, Sweden*

EXPERIMENTAL DETAILS

The number of trials for each group size ranged between 3-10 (see Table S I). Because of the large numbers of fish we used for the experiment, we reused fish between trials. Fish were never used more than once per day and fish were used a maximum of 5 times.

DATA COLLECTION AND ACQUISITION

Films were recorded in .mov format using original camera manufacturer software and subsequently converted to .avi using DirectShowSource and VirtualDub (v 1.9.2). The tracking was performed using DIDSON tracking program [1]. The raw data consisted of x and y coordinates, fish identity and a time stamp. The accuracy of the tracking process was checked by projecting the raw tracking data onto experimental videos.

MOTION STATISTICS

We characterised the degree of polarisation in the experiments and simulations by the polar order parameter [2, 3]

$$\varphi = \left\langle \frac{1}{N} \left| \sum_{i=1}^{N} \exp(\imath\theta_i) \right| \right\rangle , \tag{1}$$

where $\imath$ is the imaginary unit and θ_i is the direction of motion of individual fish (or a particle in simulations).

SELF-PROPELLED PARTICLE MODEL

In our two-dimensional self-propelled particle (SPP) model, N point particles move with a variable speed v_i at number density ρ and a time step Δt. The direction of motion of each particle (see Fig. S1) is affected by repulsive or aligning interactions with other particles located inside the zone of repulsion (zor) or zone of alignment (zoa), respectively. First, the position of each particle ($\mathbf{r}_i$) is compared to the location of other agents (Fig. S1). If other individuals are detected within zor, the focal particle attempts to keep its personal space and moves away from the neighbours. This repulsion rule has an absolute priority in the model and is modelled as a typical collision avoidance

Group size	Average body length	
	7.5 mm (small fish)	13 mm (large fish)
10	4	10
20	4	8
30	4	8
40	3	9
50	3	6
60	3	6

Table S I. Number of videos recorded for each group size and body size. Each video was 15-20 min long.

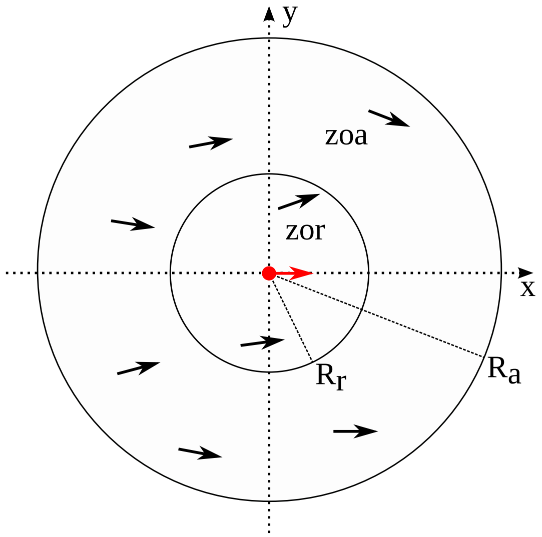

Fig. S 1. The illustration of the interaction parameters in the SPP model. The particle shown in red turns away from the nearest neighbours within the zone of repulsion (zor) to avoid collisions and aligns itself with the neighbours within the zone of alignment (zoa).

[4–6]

$$\hat{\mathbf{u}}(t)_i = -\sum_{j\neq i}^{n_r} \mathbf{r}_{ij}(t) \Big/ \left| \sum_{j\neq i}^{n_r} \mathbf{r}_{ij}(t) \right|, \tag{2}$$

with $\mathbf{r}_{ij} = \mathbf{r}_j - \mathbf{r}_i$ and n_r being a number of particles inside *zor*. If the zone of repulsion contains no neighbours, then the focal particle will respond to particles in the next zone (zoa). The alignment rule is similar to one used in the Vicsek model [2] and takes into account the velocities of all particles located inside the zoa

$$\hat{\mathbf{u}}(t)_i = \sum_{j=1}^{n_a} \mathbf{V}_j(t) \Big/ \left| \sum_{j=1}^{n_a} \mathbf{V}_j(t) \right|, \tag{3}$$

with n_a being a number of particles inside *zoa*. The velocities of the particles are then updated according to

$$\mathbf{V}_i(t) = v_i(t)\hat{\mathbf{u}}(t)_i R_1(\xi_i(t)) R_2(\theta_i(t)) \tag{4}$$

with $v_i(t) = v_0(\psi(t))^\gamma$ defining the particle's individual speed $v_i(t)$ based on the averaged local order $\psi(t)$ inside both behavioural zones [7–10]. $v_i(t)$ takes its maximal value $v_i(t) = v_0$ when the velocities of the particles inside the zor and zoa are perfectly aligned $\psi(t) = 1$ while absence of local order $\psi(t) = 0$ results in $v_i(t) = 0$. The exponent γ controls the sharpness of the speed change. The misaligning noise is introduced through a random rotation $R_1(\xi_i(t))$ of the resulting particle velocity according to a Gaussian distribution

$$P(\xi_i(t)) = e^{-\xi_i^2(t)/2\eta^2} / \sqrt{2\xi_i(t)}\eta, \tag{5}$$

where $\xi_i(t)$ is a random variable and η is the noise strength.

Wall avoidance is modelled as a particle orientation adjustment through rotation $R_2(\theta_i(t))$ of the particle velocity with a time-dependent turning rate $\theta_i(t) = v_0\phi_i(t)/d_i(t)$. $\phi_i(t)$ is the angle between the heading of a fish and the normal to a time-dependent point of impact on the wall [11, 12]. $d_i(t)$ denotes a distance from particle i to the impact point. By constructing the turning rate to the wall this way, there is strong damping of turning angles at large distances from the wall and for small angles of approach to a collision point on the wall. At these conditions, the wall's influence on the particles' motion is minimal.

The positions of particles are updated simultaneously according to

$$\mathbf{r}_i(t+\Delta t) = \mathbf{r}_i(t) + \mathbf{V}_i(t)\Delta t \tag{6}$$

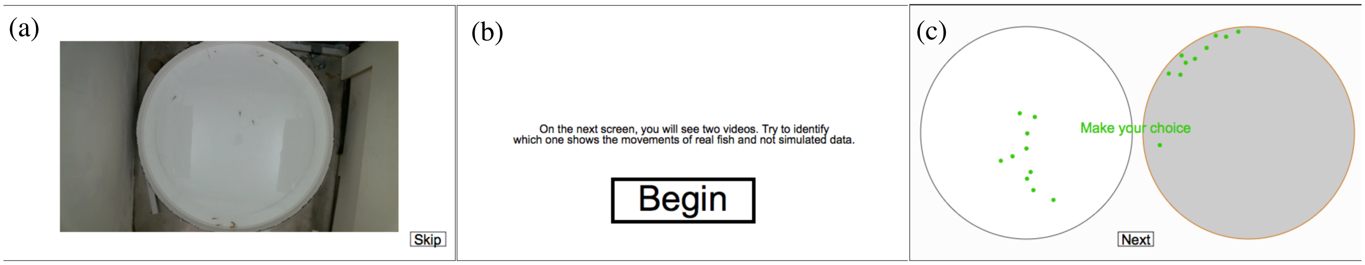

Fig. S 2. Screenshots of the web interface of the game.

The unit of length in our simulations is equivalent to the metric length used in the experiment. To set the unit of time we choose a particle speed $v_0 = bv_0^e$, where b is the behavioural reaction time [13] of fish ($b = 0.05$ s) and v_0^e is the average speed in experiment. The integration time step was set to $\Delta t = 1$ for all simulations. The noise amplitude was seto to $\eta = 0.1$ for all simulations. The differences in body size of fish were introduced through a scaling of the radius of the alignment zone proportional to the experimentally observed distribution of fish size. The radius of the repulsion zone was fixed at $R_r = 10$ for all trials. The radius of the zone of alignment was set to $R_a = 30$ and $R_a = 52.5$ for small and large fish, respectively.

INTERFACE AND STRUCTURE OF THE GAME

The graphical interface and the main module of the test were written in Processing language (`http://www.processing.org`) in JavaScript mode providing good cross-platform compatibility. The module for user statistics collection was coded in php and JavaScript. The online players statistics used in this study were collected between 12th October - 10th December 2014. We identified the number of unique IP addresses and stored the users' scores and time stamps. Statistics for the experts were collected on the 9th October 2014. Figure 2(a) shows a screenshot of the second screen of the game (the screen after the welcome screen). This screen shows a 15-second video of one of the experiments of the real fish. This is to familiarise the player with the movements of real fish. The next screen reminds the player of the rules of the game (Fig. 2(b)) and on clicking the 'Begin' button, the test starts (see Fig. 2(c) for a typical snapshot). The videos play for 5 seconds, after which they stop, and the player has to make their choice by clicking on one of the two windows. The window (left or right) that contained the simulated tracks (or trajectories from real fish) was randomised for each question. Both simulated and experimental videos were played at 15 frames per second. The 5 second sequences to be played were chosen by selecting a random starting frame in the interval 1-5300. The total length of both movies (simulated or real trajectories) used for the tests was 6 minutes. Between 7th November and 10th December 2014, if players chose the real fish correctly on all 6 questions, they were given the opportunity to provide anonymous feedback on how they differentiated between the real fish and the simulated schools using an online form.

---

* authors contributed equally

[1] Handegard NO, Williams K, 2008 Automated tracking of fish in trawls using the didson (dual frequency identification sonar). *ICES J. Mar. Sci.* **65**, 636–644. doi:10.1093/icesjms/fsn029

[2] Vicsek T, Czirók A, Ben-Jacob E, Cohen I, Shochet O, 1995 Novel type of phase transition in a system of self-driven particles. *Phys. Rev. Lett.* **75**, 1226–1229

[3] Chaté H, Ginelli F, Grégoire G, Raynaud F, 2008 Collective motion of self-propelled particles interacting without cohesion. *Phys. Rev. E* **77**, 046113

[4] Couzin ID, Krause J, James R, Ruxton GD, Franks NR, 2002 Collective memory and spatial sorting in animal groups. *J. Theor. Biol.* **218**, 1–11

[5] Couzin ID, Krause J, Franks NR, Levin SA, 2005 Effective leadership and decision-making in animal groups on the move. *Nature* **433**, 513–516

[6] Romenskyy M, Lobaskin V, 2013 Statistical properties of swarms of self-propelled particles across the order-disorder transition. *Eur. Phys. J. B* **86**, 91

[7] Li W, Wang X, 2007 Adaptive velocity strategy for swarm aggregation. *Phys. Rev. E* **75**, 021917. doi: 10.1103/PhysRevE.75.021917

[8] Zhang J, Zhao Y, Tian B, Peng L, Zhang HT, Wang BH, Zhou T, 2009 Accelerating consensus of self-driven swarm via adaptive speed. *Physica A: Statistical Mechanics and its Applications* **388**, 1237 – 1242. doi: http://dx.doi.org/10.1016/j.physa.2008.11.043

[9] Mishra S, Tunstrøm K, Couzin ID, Huepe C, 2012 Collective dynamics of self-propelled particles with variable speed. *Phys. Rev. E* **86**, 011901. doi:10.1103/PhysRevE.86.011901

[10] Lu S, Bi W, Liu F, Wu X, Xing B, Yeow EKL, 2013 Loss of collective motion in swarming bacteria undergoing stress. *Phys. Rev. Lett.* **111**, 208101. doi:10.1103/PhysRevLett.111.208101

[11] Gautrais J, Jost C, Soria M, Campo A, Motsch S, Fournier R, Blanco S, Theraulaz G, 2009 Analyzing fish movement as a persistent turning walker. *J. Math. Biol.* **58**, 429–445. doi:10.1007/s00285-008-0198-7

[12] Gautrais J, Ginelli F, Fournier R, Blanco S, Soria M, Chaté H, Theraulaz G, 2012 Deciphering interactions in moving animal groups. *PLoS Comput. Biol.* **8**, e1002678. doi:10.1371/journal.pcbi.1002678

[13] Domenici P, Blake RW, 1997 The kinematics and performance of fish fast-start swimming. *J. Exper. Biol.* **200**, 1165–1178
